# Tissue‐specific expression differences in Ras‐related GTP‐binding proteins in male rats

**DOI:** 10.14814/phy2.15928

**Published:** 2024-01-31

**Authors:** Gregory N. Kincheloe, Paul A. Roberson, Leonard S. Jefferson, Scot R. Kimball

**Affiliations:** ^1^ Department of Cellular and Molecular Physiology Penn State College of Medicine Hershey Pennsylvania USA; ^2^ Present address: Department of Anatomy UCSF College of Medicine San Francisco California USA; ^3^ Present address: Division of Endocrinology, Metabolism, Diabetes, Department of Medicine University of Colorado – Anschutz Medical Campus Aurora Colorado USA

**Keywords:** amino acid signaling, mechanistic target of rapamycin in complex 1, Rag GTPases, RagB isoform, Ras‐related GTP‐binding proteins

## Abstract

The protein kinase Mechanistic Target of Rapamycin (mTOR) in Complex 1 (mTORC1) is regulated in part by the Ras‐related GTP‐binding proteins (Rag GTPases). Rag GTPases form a heterodimeric complex consisting of either RagA or RagB associated with either RagC or RagD and act to localize mTORC1 to the lysosomal membrane. Until recently, RagA and RagB were thought to be functionally redundant, as were RagC and RagD. However, recent research suggests that the various isoforms differentially activate mTORC1. Here, the mRNA expression and protein abundance of the Rag GTPases was compared across male rat skeletal muscle, heart, liver, kidney, and brain. Whereas mRNA expression of RagA was higher than RagB in nearly all tissues studied, RagB protein abundance was higher than RagA in all tissues besides skeletal muscle. RagC mRNA expression was more abundant or equal to RagD mRNA, and RagD protein was more abundant than RagC protein in all tissues. Moreover, the proportion of RagB in the short isoform was greater than the long in liver, whereas the opposite was true in brain. These results serve to further elucidate Rag GTPase expression and offer potential explanations for the differential responses to amino acids that are observed in different tissues.

## 1 INTRODUCTION

Mammals contain many different types of tissues, which can sometimes react in a different manner to various nutrients and anabolic stimuli. These differing responses are often associated with differential activation of the protein kinase Mechanistic Target of Rapamycin (mTOR) when it is present in Complex 1 (mTORC1). Often referred to as the master regulator of protein synthesis, it is an essential player in the initiation of mRNA translation, and it is activated by upstream signals generated both by nutrients, such as leucine and glucose, as well as by growth factors such as insulin and insulin‐like growth factor 1 (IGF1) (Roberson et al., 2023; Saxton & Sabatini, 2017). When activated, mTORC1 phosphorylates downstream targets that promote protein synthesis, such as Eukaryotic Initiation Factor 4E Binding Protein 1 (4E‐BP1) and p70 Ribosomal S6 Kinase 1 (p70S6K1). mTORC1 also phosphorylates transcription factors such as Transcription Factor EB and E3 (TFEB and TFE3, respectively), preventing them from entering the nucleus and promoting lysosome biogenesis (Gollwitzer et al., 2022; Kim & Kim, 2016).

Canonically, mTORC1 is activated at the lysosomal membrane by the small GTPase Rheb when the GTPase‐activating‐protein (GAP) activity of the tuberous sclerosis complex (TSC) is inhibited by hormones and growth factors such as insulin and IGF‐1, thereby preventing the conversion of active Rheb‐GTP to inactive Rheb‐GDP (Kim & Kim, 2016; Kimball et al., 1997; Roberson et al., 2022; Yang et al., 2017). Importantly, mTORC1 localization to the lysosomal membrane relies on a heterodimeric complex consisting of two Rag GTPases formed from either a smaller RagA or RagB in association with a larger RagC or RagD (Kim & Kim, 2016). When RagA/B is GTP‐loaded and RagC/D is GDP‐loaded, they localize to the lysosomal membrane by binding to the Ragulator complex, a five‐protein lysosomal membrane‐bound complex formed by LAMTOR1‐5. Binding of a Rag heterodimer to both mTORC1 and Ragulator serves to bring mTORC1 to the lysosomal membrane where it interacts with Rheb in a process that is required for mTORC1 activation (Bar‐Peled et al., 2012; Kim & Kim, 2016; Sancak et al., 2008, 2010).

Until recently, the RagA/B isoforms were considered to be functionally redundant, as were the RagC/D isoforms, though small differences among these complexes, such as leucyl‐tRNA synthetase interacting with RagD but not RagC as well as an extra phosphorylation site on RagC, were observed in cells that hinted at potential functional variances (Han et al., 2012; Yang et al., 2019). However, more recent research has indicated that heterodimers containing RagC and RagD differ in that RagD‐containing heterodimers are more effective at localizing mTORC1 to the lysosomal membrane as well as leading to increased phosphorylation of TFEB and TFE3 compared to RagC‐containing heterodimers (Gollwitzer et al., 2022). In addition, RagB‐containing heterodimers have been shown to be more resistant to amino acid deprivation than RagA‐containing heterodimers (Figlia et al., 2022; Gollwitzer et al., 2022). Expression of Rag mRNAs has also hinted at potential tissue‐specific differences in these Rag‐GTPases, such as RagD expression being dominant to RagC in skeletal muscle and heart, whereas the opposite is true in many other tissues, for example, liver (Figlia et al., 2022; Gollwitzer et al., 2022). Though mRNA expression has been investigated, protein abundance of the different Rag isoforms may differ from that of mRNA and further reveal tissue‐specific phenotypes. Given that mRNA expression often correlates poorly with protein abundance (Buccitelli & Selbach, 2020), in the present study the specificity of Rag GTPase mRNA and protein abundance in five rat tissues was investigated to shine further light on the potential effectors of the differing anabolic responses to stimuli that are observed across organ systems.

## 2 MATERIALS AND METHODS

### 2.1 Animal protocol

All rats utilized in this study were housed in temperature‐controlled rooms on a 12:12 light: dark schedule. Male Sprague–Dawley rats weighing between 150 and 175 g were purchased from Charles River Laboratories (RRID:RGD_737891) and underwent at least 1 week of acclimation in the animal facility before experimental protocols began. Experimental procedures were approved by the Institutional Animal Care and Use Committee of Penn State College of Medicine following National Institutes of Health guidelines. Rats were given food and water ad libitum until the onset of experimental procedures and subsequent collection of tissue samples.

### 2.2 Experimental design

#### 2.2.1 Rat tissue collection

Tissues were collected approximately 3 h after the lights came on. Before collecting tissues, rats were anesthetized with 2%–3% isoflurane in O_2_ to induce a proper surgical plane. To maximize blood perfusion to organs before collection, tibialis anterior muscle was collected first, followed by kidney, liver, heart (apex), and then brain. Prior to analyzing the Rag protein expression in each of the tissues, relative Rag mRNA values for the gastrocnemius (a mixed‐fiber muscle) and tibialis anterior (a predominantly fast‐twitch muscle) were assessed and found to be comparable (data not shown), and thus tibialis anterior was chosen for all subsequent experiments for the purpose of minimizing blood loss and timing to collect the rest of the organs. Tissues were immediately homogenized with plastic pestles in either Trizol reagent (Invitrogen #15596026) for subsequent mRNA analysis or ice‐cold homogenization buffer containing 20 mM HEPES, pH 7.4, 2 mM EGTA, 50 mM NaF, 100 mM KCl, 0.2 mM EDTA, 50 mM β‐glycerophosphate, 2 mM DTT, 1 mM sodium vanadate, 2 mM benzamidine, and 30 μL/mL protease inhibitor cocktail (Sigma Aldrich, #P8340). Tissue homogenates were then centrifuged at 13,400×g at 4°C for 10 min, the supernatant was collected, and protein content was quantified using Bradford assays (Bio‐Rad, #5000006). Samples were standardized for protein and then diluted 1:2 with 4X SDS buffer (0.14 M Tris–HCl, 28% glycerol, 2.8% SDS, 0.22% bromophenol blue, pH 6.8) and β‐mercaptoethanol (BME) and then boiled at 100°C for 5 min.

#### 2.2.2 Western blotting

Rat tissue protein samples (50 μg of protein/well) were prepared as described above and separated by SDS‐PAGE on 4%–20% Criterion TGX precast gels (#5671095 Bio‐Rad, Hercules, CA). Proteins were then transferred to PVDF membranes (Millipore #IPV00010), and the membranes were stained for total protein using Ponceau S (Sigma‐Aldrich #P3504) before being blocked in 5% nonfat milk dissolved in Tris‐buffered saline containing 0.1% Tween‐20 (TBST) (Sigma‐Aldrich #P1379) for 1 h at room temperature. Membranes were then probed with antibodies to either RagA (Cell Signaling Technology #4357, RRID:AB_10545136; 1:1000), RagB (Proteintech #13023‐1AP, RRID:AB_2180064; 1:1000), RagC (Cell Signaling Technology #3360, RRID:AB_2180068, 1:1000), or RagD (Mybiosource #MBS9201281, 1:1000) at 4°C overnight. Of note, the RagD antibody recognized two splice variants of RagD that flanked the 50 kDa marker. Though no such splice variants have currently been reported in rats, mice have two RagD variants with estimated molecular weights of 51 kDa and 45 kDa (Zhang et al., 2021). In addition, shRNA knockdown of RagD in H4IIE rat hepatoma cells (ATCC, CRL‐1548, RRID:CVCL_0284; passage 11) resulted in a reduction in both bands (Figure S1). As a result, we quantified both RagD bands in the present study. The next day, after washing thrice for 5 min in TBST, membranes were incubated for 1 h with anti‐rabbit HRP‐conjugated secondary antibodies (Cell Signaling #7074, 1:3,000; RRID:AB_2099233) in 5% milk at room temperature. After washing again in TBST, membranes were covered in Clarity Western ECL Blotting Substrate (Bio‐Rad #1705060) before subsequent imaging on a Fluorchem M imaging system (ProteinSimple, San Jose, CA). Blots were analyzed using ImageJ (NIH, Bethesda MD; RRID:SCR_003070) (Kincheloe et al., 2023).

#### 2.2.3 Rag protein purification

To purify Rag proteins for use in generating standard curves for quantification of Rag protein abundance in rat tissues, HEK293T cells were plated on 150 mm plates (Sigma‐Aldrich #639160), and transfected at 80%–90% confluency using Lipofectamine 2000 (ThermoFisher #11668019) and 24.4 μg of pCMV‐3Tag‐1A plasmid expressing FLAG‐tagged rat Rag proteins: 3xFLAG‐RagA, 3xFLAG‐RagB, 3xFLAG‐RagC, or 3xFLAG‐RagD (GenScript, Piscataway, NJ). Forty‐eight hours after transfection, 50 μL of Anti‐FLAG M2 Magnetic Beads (Sigma‐Aldrich #M8823; RRID:AB_2637089) per 150 mm plate of transfected cells were washed three times in five volumes of CHAPS wash buffer (40 mM HEPES, pH 7.5, 120 mM NaCl, 1 mM EDTA·Na_2_, 10 mM sodium pyrophosphate, 10 mM β‐glycerophosphate disodium salt hydrate, 50 mM NaF, 0.3% CHAPS (3‐((3‐ cholamidopropyl)dimethylammonio)‐1‐propanesulfonate)) before being incubated at 4°C in CHAPS lysis buffer (40 mM HEPES, pH 7.5, 120 mM NaCl, 1 mM EDTA·Na_2_, 10 mM sodium pyrophosphate, 10 mM β‐glycerophosphate disodium salt hydrate, 50 mM NaF, 0.3% CHAPS, 1 mM microcystin‐LE, 200 mM sodium vanadate, and 10 μL/mL protease inhibitor cocktail (Sigma‐Aldrich #P8340)) for 1 h on an agitator (Roberson et al., 2023; Xu et al., 2019). While the anti‐FLAG beads were incubating, transfected cells were washed once in ice‐cold PBS, lysed in CHAPS lysis buffer, and centrifuged at 13,400×g at 4°C for 10 min after which the supernatant was transferred to a fresh tube. After the hour of incubation in CHAPS lysis buffer, anti‐FLAG M2 Beads were added to the cell lysate and incubated at 4°C for 2 h with agitation. Beads were then washed four times in high salt buffer (40 mM HEPES, pH 7.4, 500 mM NaCl, 2.5 mM MgCl_2_, 1% Triton X‐100) followed by two 30‐min washes in 50 μL 3xFLAG peptide (150 ng/μL, Sigma‐Aldrich #F‐4799) at 4°C to elute the FLAG‐tagged protein (Xu et al., 2019). These washes were subsequently pooled before each purified sample was diluted in an equal volume of 8 M urea dissolved in CHAPS lysis buffer. Samples were then adjusted to 8 M urea using Slide‐A‐Lyzer™ MINI Dialysis Devices (ThermoFisher #69576) and incubated for 10 min at 30°C to solubilize any insoluble Rag protein. Protein content was quantified using a NanoOrange Protein Quantification Kit (Invitrogen #N‐6666) and standardized to RagA values observed from SimplyBlue Safestain (Invitrogen # LC6060). Rag protein standard curves were created for each of the four Rag proteins based on these adjusted concentrations, and the curves were used to calculate the abundance of each Rag protein in the five tissues examined in this study.

#### 2.2.4 mRNA isolation and qPCR

Rat tissue samples were collected and homogenized in Trizol reagent as described above, and then underwent mRNA isolation following the manufacturer's protocol. After quantification using a NanoDrop 1000 Spectrophotometer (ThermoFisher Scientific, Waltham MA; RRID:SCR_016517), 1 μg of mRNA was used to produce cDNA using the High‐Capacity cDNA Reverse Transcription Kit (Applied Biosystem #4374966) following the manufacturer's protocol. After cDNA was produced, qPCR was performed in triplicate with SYBR Green Master Mix (Qiagen #204143) and primers (Integrated DNA Technologies) for Rat β‐actin (Forward 5′‐CAGGGTGTGATGGTGGGTATGG‐3′, Reverse 5′‐AGTTGGTGACAATGCCGTGTTC‐3′), Rat RagA (Forward 5′‐CGGGACAACATCTTCCGTAAT‐3′, Reverse 5′‐GCTTCCAGACACGACTGATAAT‐3′), Rat RagB (Forward 5′‐GACTAAGTTCGGGTCGGTAAAG‐3′, Reverse 5′‐GTTCTTCTGGCTCACCTTCTC‐3′), Rat RagC (Forward 5′‐CCCTGTGGATATGCAGTCTTAC‐3′, Reverse 5′‐AGCACTTCCACTTCCATCTTC‐3′), and Rat RagD (Forward 5′‐GCCTACAAGGTGAACACTGATA‐3′, Reverse 5′‐CCCTCTGGTGAATGTCTCTTT‐3′). mRNA was normalized to β‐actin and analyzed utilizing the 2^−ΔCT^ method (Schmittgen & Livak, 2008).

#### 2.2.5 Statistical analysis

Statistical analysis was performed using GraphPad Prism 9.0.2 (La Jolla, CA; RRID:SCR_002798). Data are shown as the mean ± SD. One outlier more than two standard deviations from the mean was omitted when calculating the RagC/RagD protein abundance ratio for liver as noted in the legend to Figure 3. In comparing tissue‐specific expression of Rag GTPase proteins, a one‐way ANOVA was used with Bonferroni post hoc multiple comparisons utilized when a significant interaction was identified. The significance was set at *p* < 0.05. Thresholds for trending data were set at 0.05 < *p* < 0.1.

## 3 RESULTS

### 3.1 Tissue distribution of Rag mRNA expression in rats

**FIGURE 1:**
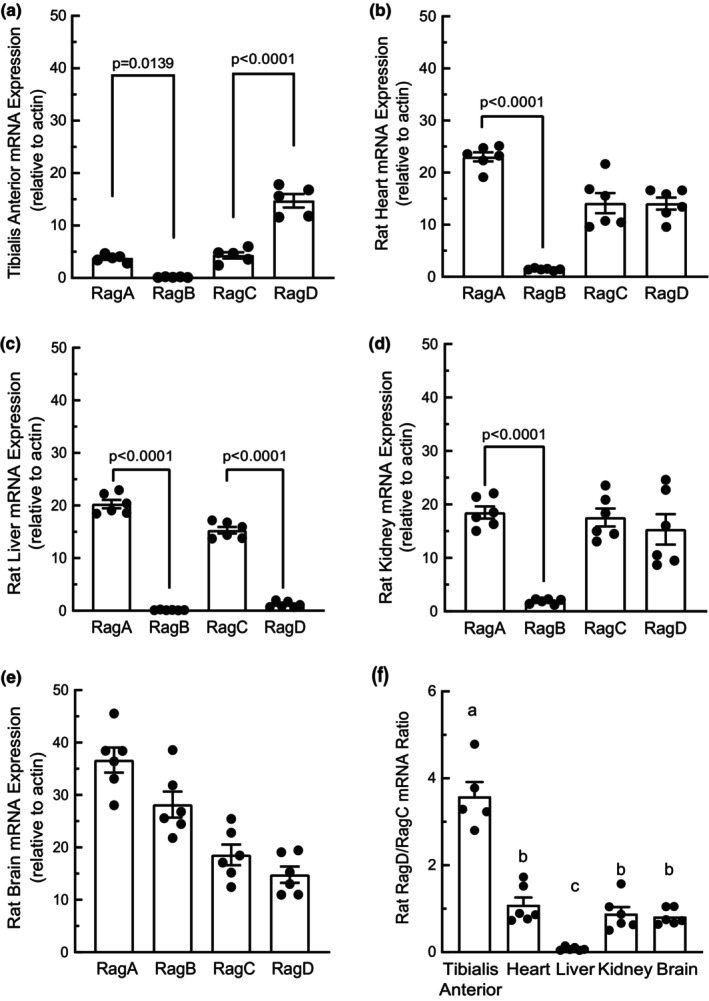
Rag GTPase mRNA expression in rat tissues. Rag GTPase mRNA Expression in rat (a) tibialis anterior skeletal muscle, (b) heart, (c) liver, (d) kidney, and (e) brain. *N* = 5–6; ** *p* < 0.01. (f) RagD/RagC mRNA ratios in each of the five rat tissues. *N* = 6; bars labeled with different letters are significantly different, *p* < 0.05.

We collected tibialis anterior skeletal muscle, heart, liver, kidney, and brain tissue and subsequently measured Rag mRNA expression in each. Rat tissues expressed predominantly RagA mRNA rather than RagB mRNA, with the exception of brain in which RagA mRNA and RagB mRNA expressions were not significantly different (Figure 1a–e). Notably, RagA mRNA expression in liver was 150‐fold higher compared to RagB, resulting in liver having the highest RagA/RagB mRNA ratio (Figure 1c). Rat skeletal muscle expressed significantly more RagD mRNA than RagC mRNA, whereas in the heart, kidney, and brain RagC and RagD mRNAs were expressed at similar levels, and in liver the RagC mRNA expression was significantly higher compared to RagD mRNA (Figure 1f).

### 3.2 Tissue distribution of Rag protein abundance in rats

**FIGURE 2:**
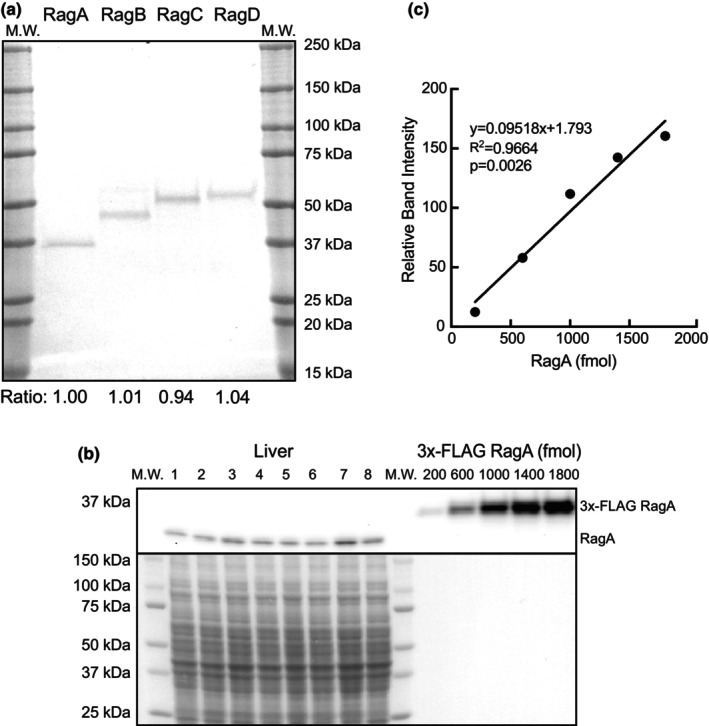
Rat Rag protein abundance and purification. (a) 3X‐FLAG‐tagged rat RagA, RagB, RagC, and RagD proteins were purified as described under Materials and Methods. After quantification, 50 pmol of each purified 3x‐FLAG‐tagged Rag protein was resolved by SDS‐PAGE and the gel was stained with SimplyBlue Safestain. Values below the image are density ratios expressed relative to RagA. (b) *top panel*: Representative RagA blot of 50 μg of liver protein from eight different rats and varying amounts of 3X‐FLAG‐RagA. *Bottom panel*: Total protein abundance assessed via Ponceau S stain of the PVDF membrane prior to western blot. M.W., molecular weight standards. (c) Representative 3X‐FLAG‐RagA standard curve utilized to quantify tissue Rag GTPase abundance in each western. The processes shown in 2B‐2C were performed for each individual Rag protein isoform in each tissue to ascertain protein abundance data.

**FIGURE 3:**
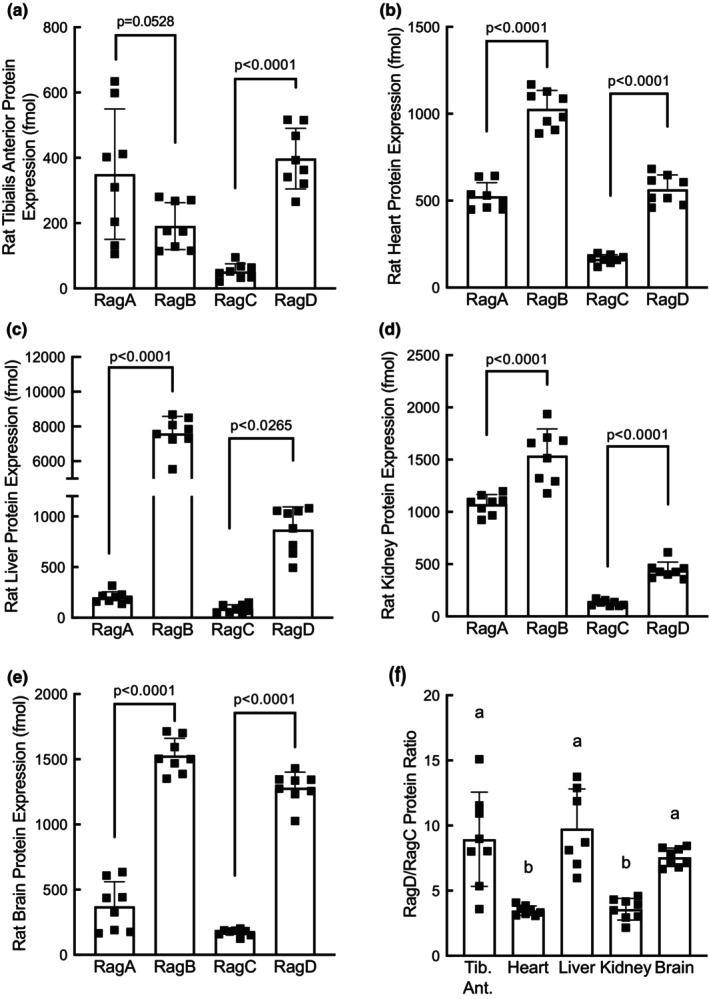
Rag GTPase protein abundance in rat tissues. Rag GTPase protein abundance in rat (a) tibialis anterior, (b) heart, (c) liver, (d) kidney, and (e) brain. *N* = 8. (f) RagD/RagC protein ratio in tibialis anterior, heart, liver, kidney, and brain. Tib. Ant., tibialis anterior. One value for liver was more than two standard deviations from the mean and was excluded from the analysis. *N* = 7–8, bars labeled with different letters are significantly different.

To assess Rag GTPase protein abundance across tissue types, plasmids expressing 3x‐FLAG‐tagged variants of each of the Rag proteins were transfected into HEK293T cells, and the expressed proteins were affinity purified using anti‐FLAG beads to near homogeneity (Figure 2a). Serial dilutions of the purified Rag proteins were then used to create standard curves to quantify tissue differences in Rag protein abundance. As an example of the process that was performed for all Rag proteins in each of the tissues, Figure 2b,c show the quantification of RagA in rat liver extracts. These analyses showed that, contrary to the results of the mRNA expression analyses, RagB protein abundance was higher than RagA protein abundance in heart, liver, kidney, and brain tissue (Figure 3b–e). Interestingly, the opposite pattern was observed in skeletal muscle, that is, there was a trend for RagA protein abundance to be higher than RagB (Figure 3a). Additionally, it is notable that the liver expressed 10‐fold more RagB protein than RagA protein, the most of any of the other tissues (Figure 3c). Also contrary to the mRNA expression results, RagD protein abundance was significantly higher than RagC in all the tissues (Figure 3a–e). Analysis of the RagD protein abundance compared to RagC across tissues showed a significantly higher RagD/RagC ratio in skeletal muscle, liver, and brain compared with heart and kidney (Figure 3f).

### 3.3 RagB protein isoform abundance correlates with mTORC1 sensitivity to leucine administration

**FIGURE 4:**
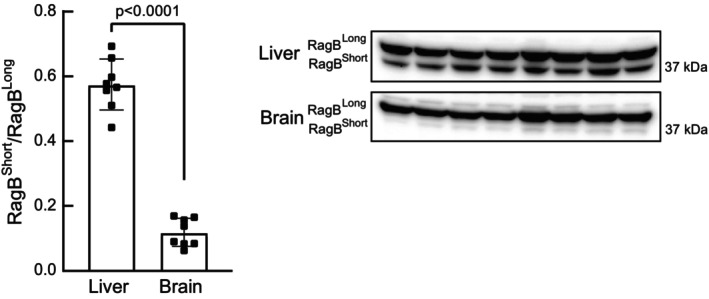
RagB^Short^/RagB^Long^ isoform abundance in liver and brain. The distribution of RagB in the short and long isoforms in liver and brain was assessed by Western blot analysis. The ratio of RagB^short^/RagB^long^ is shown in the graph. *N* = 8.

We previously showed that oral administration of leucine to fasted rats robustly increased mTORC1 signaling in the liver, with little or no activation in brain (Xu et al., 2019). A recent study (Figlia et al., 2022) showed that RagB exists in two isoforms, referred to as RagB^short^ and RagB^long^. Interestingly, mTORC1 in cells expressing just the RagB^long^ isoform is completely insensitive to differences in amino acid availability, whereas in cells expressing the RagB^short^ isoform mTORC1 activity is repressed when cells are deprived of amino acids while mTORC1 activity is elevated in cells maintained in amino acid‐replete medium. Based on these results, we hypothesized that the proportion of RagB in the short isoform would be higher in liver compared with brain, and the opposite would be true for the RagB^long^ isoform. Indeed, as shown in Figure 4, brain expresses primarily RagB^long^ whereas, in comparison to brain, the liver has a significantly higher proportion of RagB^short^.

## 4 DISCUSSION

In a previous study using RNAseq analysis to assess mRNA expression in a variety of human tissues, RagA mRNA was shown to be more highly expressed than RagB mRNA in skeletal muscle, heart, liver, kidney, and brain (Uhlen et al., 2015). Moreover, although the RagC and RagD mRNAs were expressed at similar levels in the human liver and brain, in skeletal muscle, heart, and kidney, RagD mRNA expression was higher than RagC mRNA. The results of the present study assessing Rag isoform mRNA expression in rat tissues show similarities and dissimilarities with those of the previous study. For example, although RagA mRNA expression was higher than RagB mRNA in rat skeletal muscle, heart, liver, and kidney, the two mRNAs were expressed at similar levels in rat brain. If protein abundance is directly proportional to mRNA expression, then RagA protein abundance should be higher than RagB protein in tissues other than brain where the two proteins should be expressed at similar levels. Moreover, RagD protein abundance in skeletal muscle should be higher than RagC protein abundance, lower in liver, and similar in the other three tissues examined. However, recent studies suggest that only about 40% of the variability in protein abundance is explained by variability in mRNA expression (Buccitelli & Selbach, 2020; reviewed in: 14). Therefore, in the present study, Rag protein abundance was also assessed.

In agreement with the mRNA expression data, there was a strong trend for RagA protein abundance to be higher than RagB protein abundance in rat skeletal muscle. Similarly, RagD protein abundance was higher than RagC protein in that tissue. However, in the other four tissues that were examined, mRNA expression was a poor predictor of protein abundance. For example, rather than being lower than RagA as was observed for mRNA expression, RagB protein abundance was higher than RagA protein in heart, liver, kidney, and brain. Discrepancies between mRNA expression and protein abundance can be mediated by differences in the rate at which a given mRNA is translated into protein, variations in rates of protein degradation, or both. Although assessing rates of Rag mRNA translation and Rag protein turnover are beyond the scope of the present study, it is interesting that the 5′‐untranslated regions (5′UTR) of each of the Rag mRNAs have potential regulatory motifs that might play important roles in modulating their translation. For example, the guanine‐cytosine content of the 5′UTR of the RagA mRNA is nearly 10% higher than that of RagB, which may cause less efficient scanning and lower initiation rates (Leppek et al., 2018). Also, the RagD 5′UTR contains an uninterrupted stretch of seven pyrimidines immediately distal to the 5′‐m^7^GTP cap, which could serve as a Terminal Oligopyrimidine (TOP)‐like motif, linking translation to the energy status of the cell and increasing translation when mTORC1 is activated (Cockman et al., 2020). In freely‐fed rats, as utilized in the present study, this may explain the greater abundance of RagD protein compared with RagC protein observed in most tissues.

The roles of the various Rag heterodimers in the regulation of mTORC1 by amino acids were assessed in a recent study in which all four Rag isoforms were knocked out in HEK293FT cells (Gollwitzer et al., 2022). RagD was subsequently coexpressed in the quad knockout cells in combination with either RagA or RagB to form RagA/D or RagB/D heterodimers. Alternatively, RagA was coexpressed with either RagC or RagD to form RagA/C or RagA/D heterodimers. Downregulation of mTORC1 activity in response to amino acid deprivation, as assessed by phosphorylation of its direct target p70S6K1 on Thr389, occurred to a similar extent in cells expressing either the RagA/D or RagB/D heterodimeric complexes, suggesting that when associated with RagD, RagA, and RagB act in a redundant manner to promote mTORC1‐mediated phosphorylation of p70S6K1(Thr389). By contrast, cells expressing the RagA/C heterodimer were significantly less sensitive to amino acid deprivation‐induced downregulation of mTORC1 activity compared to either wildtype cells or cells expressing the RagA/D heterodimer (Figure S2). These results suggest that RagD may play a more important role compared with RagC in signaling amino acid availability to mTORC1. However, in a previous study (Xu et al., 2019) we showed that oral leucine administration to fasted rats upregulated mTORC1 activity in all tissues examined except the brain. In the present study, RagD protein abundance was higher than RagC protein in all the tissues examined, including brain, suggesting that the difference in sensitivity of mTORC1 to oral leucine administration may not be explained solely by the abundance of those two Rag isoforms.

Results from another recent study (Figlia et al., 2022) suggest that the importance of RagB in mediating amino acid signaling to mTORC1 depends on whether the short or long isoform is expressed. For example, as observed in wildtype cells, in RagA/B double knockout cells (RagABDKO) in which either RagA or RagB^short^ is expressed, mTORC1‐mediated phosphorylation of p70S6K1 on Thr389 is high under amino acid‐replete conditions and low in cells deprived of amino acids. By contrast, mTORC1 activity is low in RagABDKO cells expressing RagB^long^ regardless of amino acid availability. In the present study, the proportion of RagB in the short isoform in liver was 58% of the total, whereas in brain the short isoform was only expressed at 12% of the level of the long isoform. Attempts to assess RagB isoform distribution in the other three tissues were unsuccessful. However, based on our previously published results (Xu et al., 2019) showing that oral leucine administration to fasted rats increased p70S6K1(Thr389) phosphorylation in the liver, but not the brain, we hypothesize that the difference in isoform RagB distribution in the two tissues may play a role in the differential activation of mTORC1. This hypothesis will need to be tested in future studies.

It should be noted that the Rags comprise just one of several signaling pathways that regulate mTORC1 activity. For example, recent studies implicated ADP ribosylation factor 1 (Arf1) and its GTPase activating protein, Arf1gap, in Rag‐independent activation of mTORC1 by the amino acids Asn and Gln (Jewell et al., 2015; Meng et al., 2020, 2021). Interestingly, Gln‐dependent activation of mTORC1 depends not only on Arf1, but also on phospholipase D, Rheb, and ras‐like proto‐oncogene A (RalA) (Bernfeld et al., 2018). Other studies showed that G‐protein coupled receptor signaling inhibits mTORC1 through protein kinase A dependent phosphorylation of the mTORC1 subunit, Raptor (Jewell et al., 2019), and thus might potentially occur in a manner independent of both Rag and Rheb. To our knowledge, the tissue distribution of most of these proteins has not been assessed, and thus, their relative contributions to the regulation of mTORC1 remains to be determined.

## 5 CONCLUSIONS

The results of the present study show that Rag mRNA expression is a poor indicator of Rag protein abundance in the rat tissues that were examined. Though the effects of the differing Rag protein abundance on tissue protein metabolism are currently speculative, they provide an avenue for future investigation and potential therapeutic targets to affect mTORC1 signaling. As dysfunction in specific Rag GTPases and their upstream regulators have been implicated in cardiomyopathy, kidney tubulopathy, epilepsy, and cancer (Iffland 2nd et al., 2019; Okosun et al., 2016; Sambri et al., 2023), identifying differences in the function of each of the different Rag GTPases and how they are expressed in various tissue types may provide avenues for future therapeutic targets for treatment of these diseases.

## FUNDING INFORMATION

This work was supported by NIH grants R01DK015658 (S.R.K.) and F32DK126312 (P.A.R.).

## CONFLICT OF INTEREST STATEMENT

The authors have no relevant disclosures or conflict of interest to declare.

## ETHICS STATEMENT

Studies involving animals in this report were approved by the Penn State College of Medicine Institutional Animal Care and Use Committee.

## Supporting information


Figure S1

Figure S2

## Data Availability

Data available on request from the authors.
