# Supplementary material for: Tissue‐specific expression differences in Ras‐related GTP‐binding proteins in male rats
Source: Physiol Rep. 2024 Jan 31;12(3):e15928. doi: 10.14814/phy2.15928 (PMC10830385; doi:10.14814/phy2.15928)
Supplement: Supplementary file 1 — Figure S1 [file PHY2-12-e15928-s001.pdf]

Supplementary Figure 1

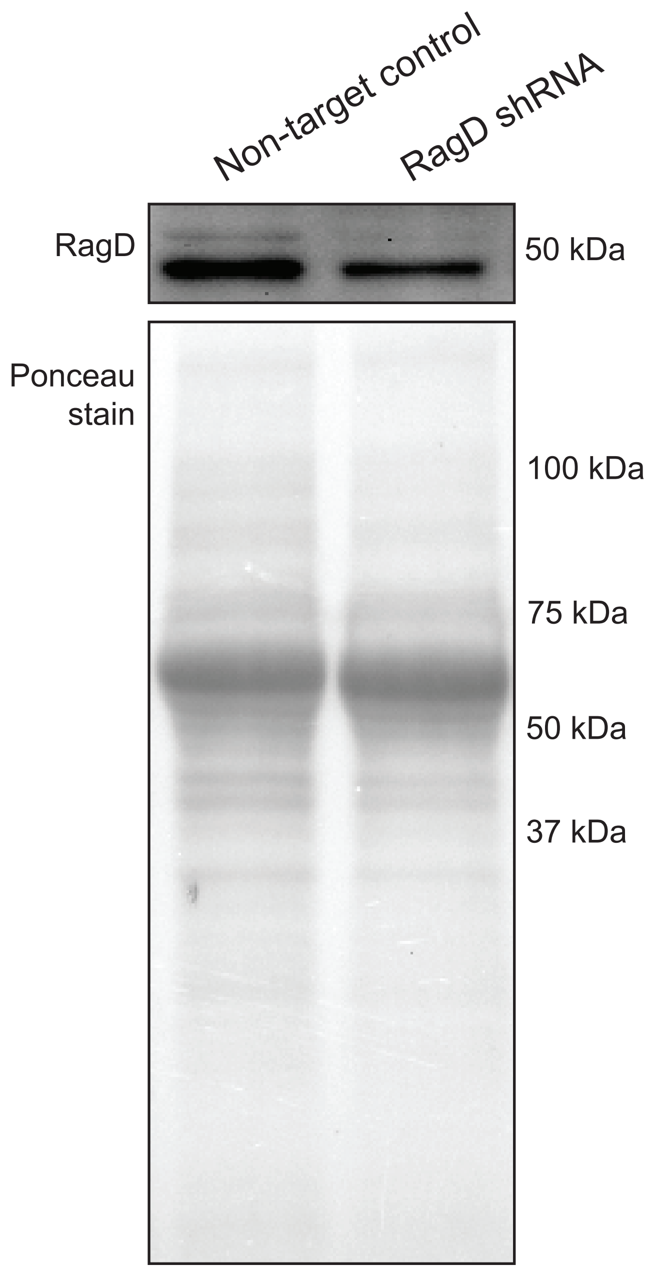

Supplemental Figure 1: shRNA knockdown of RagD. H4IIE cells were transfected with either non-target control shRNA or shRNAs targeting RagD (5'- CCGGCTTTAGCAAAGTG-GTTCAGAACTCGAGTTCTGAACCACTTTGCTAAAGTTTTTG-3' and 5'- CCGGACAAG-GACTCAACAGCCATTACTCGAGTAATGGCTGTTGAGTCCTTGTTTTTTG-3'). 24 h later, cells were harvested and subjected to Western blot analysis for RagD protein abundance Two bands were detected with molecular weights expected for the short and long splice forms of RagD. Both exhibited diminished abundance in RagD shRNA-treated cells compared to control cells.
