# Supplementary material for: Tissue‐specific expression differences in Ras‐related GTP‐binding proteins in male rats
Source: Physiol Rep. 2024 Jan 31;12(3):e15928. doi: 10.14814/phy2.15928 (PMC10830385; doi:10.14814/phy2.15928)
Supplement: Supplementary file 2 — Figure S2 [file PHY2-12-e15928-s002.pdf]

Supplementary Figure 2

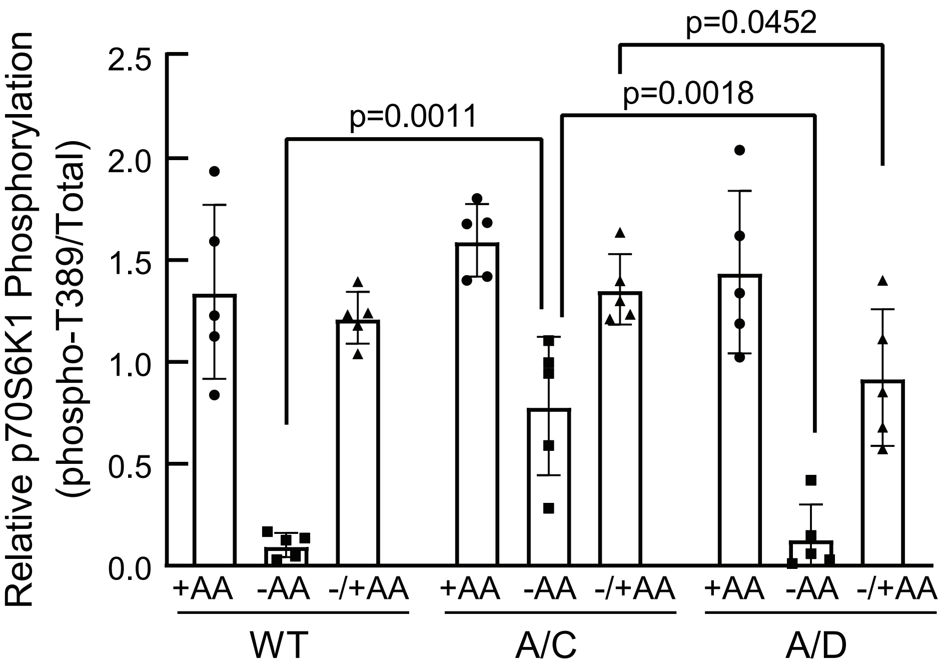

Supplemental Figure 2: Analysis of source data from Gollwitzer, et al. (doi: 10.1038/s41556-022-00976-y). mTORC1 activation in wildtype (WT) cells or RagA/B/C/D quadruple knockout cells expressing only RagA/C (AC) or RagA/D (AD) was assessed in cells maintained in complete medium (+AA), cells deprived of amino acids for 2h (-AA), and cells deprived of amino acids followed by addback of amino acids for 30 min (-/+AA). N=5; 2-way ANOVA: * p=0.045, ** p<0.01.
